# Implementation of S. aureus surveillance for prevention of blood stream infections in hemodialysis patients

**DOI:** 10.1186/2047-2994-4-S1-P64

**Published:** 2015-06-16

**Authors:** L Coucke, C Verfaillie, J Van Acker, M De Clippele, J Donck, A-M Van den Abeele

**Affiliations:** 1 Laboratory of Clinical Microbiology, Laboratory Medicine, AZ Sint-Lucas Ghent, Ghent, Belgium; 2 Nephrology Department, AZ Sint-Lucas Ghent, Ghent, Belgium

## Introduction

Surveillance for *S. aureus* (SA) nasal carriage followed by decolonization is a validated strategy to reduce infections and mortality in hemodialysis (HD) patients. This practice is not widespread in Belgian HD units. The current prevalence of methicillin sensitive SA (MSSA) and methicillin resistant SA (MRSA) carriers in our HD population is unknown.

## Objectives

To evaluate the feasibility of routine surveillance and to assess SA prevalence, a point–prevalence survey measuring the colonization rate of HD patients was organised.

## Methods

Screening of all HD patients was performed in January, June and November 2014. Home HD (n=1) and peritoneal dialysis (n=5) patients were excluded. Separate swabs (ESwab, Copan, Italy) were taken from anterior nares, wounds and the insertion site of the dialysis catheter. For MSSA detection, swabs were inoculated directly on chromogenic SA plates (SaSelect^TM^ medium, Bio–Rad Laboratories, Belgium). MRSA was isolated on chromogenic MRSA plates (BD^TM^ BBL CHROMagar MRSAII, Becton-Dickinson, Belgium) after overnight enrichment in tryptic soy broth of 100µL ESwab Amies medium. Suspected colonies were identified by gram stain and matrix–assisted laser desorption/ionization time–of–flight mass–spectrometry. MRSA confirmation was obtained by *mecA* gene and *nuc* gene polymerase chain reaction.

## Results

154 patients (mean age 70 years, range 27-95 years) from 3 HD units were screened: 1 in-hospital unit (n=107), 2 outpatient centres (n=47). Mean dialysis time was 4.2 years. 14% of patients were in residential care. SA colonization rate was stable from January to November (26.3%). MRSA prevalence was 1.3% in January/June and 2.8% in November. Outpatient centres showed similar MSSA prevalence (33% and 28%) to the in-hospital unit (17%). 13 patients (9.2%) were persistent MSSA carriers, 8 of them carrying a central line (5.6%). None of the catheter insertion site swabs demonstrated SA.

## Conclusion

Nasal MSSA prevalence in HD patients equals the general population prevalence (29%–32%), MRSA colonization rate (1.3%–2.8%) is low compared to the total in-hospital at-risk population (4%). Routine surveillance 3 times a year is achievable and should include MSSA and MRSA screening in all HD patients. New patients should be screened upon HD start.

## Disclosure of interest

None declared.

